# *Foeniculum vulgare* Miller, a New Chemotype from Montenegro

**DOI:** 10.3390/plants11010042

**Published:** 2021-12-23

**Authors:** Mijat Božović, Stefania Garzoli, Svetlana Vujović, Filippo Sapienza, Rino Ragno

**Affiliations:** 1 Faculty of Natural Sciences and Mathematics, University of Montenegro, Džordža Vašingtona bb, 81000 Podgorica, Montenegro; 2 Department of Drug Chemistry and Technology, Sapienza University, P.le Aldo Moro 5, 00185 Rome, Italy; stefania.garzoli@uniroma1.it (S.G.); filosapi@gmail.com (F.S.); 3 Institute for Medicines and Medical Devices in Montenegro, Ivana Crnojevića 64a, 81000 Podgorica, Montenegro; svetlana.vujovic@cinmed.me

**Keywords:** *Foeniculum vulgare* Miller, Apiaceae, essential oils, prolonged extraction, fractionated distillation, HS-GC/MS, *α*-terpineol, anethole, fenchone

## Abstract

Previous studies relating to prolonged and fractionated distillation procedures highlighted essential oils’ (EOs) chemical composition to be significantly dependent on the extraction duration and harvesting time. As a continuation, a hydrodistillation procedure was applied to ripe fruit material of fennel, *Foeniculum vulgare* Miller (Apiaceae), collected from three localities in Montenegro (Podgorica, Nikšić, and Kotor) to furnish a total of 12 EOs. Liquid and vapor phases of the samples were analyzed by Gas Chromatography/Mass Spectrometry and Headspace-Gas Chromatography/Mass Spectrometry techniques, and 18 compounds have been identified. Although both quantitative and qualitative differences between the samples were notable, the phenylpropanoids anethole (ANE) and estragole and the monoterpenoids *α*-terpineol (TER) and fenchone (FEN) could be singled out as the most abundant constituents. The EOs from Podgorica belong to the most common ANE-rich chemotype, while the predominance of the monoterpenoid fraction is characteristic of the samples from Nikšić and Kotor. The latter is particularly rich in TER (up to 56.5%), with significant amounts of FEN and ANE. This chemical profile could represent a new chemotype of fennel EO. Vapor phases contained mainly monoterpenoids, with increased amounts of FEN and TER, while the number of phenylpropanoids was significantly decreased.

## 1. Introduction

As aromatic and volatile liquids obtained from different plants and their different parts, essential oils (EOs) have been regarded with great interest throughout human history [1]. Many of them and/or their ingredients have an unexpectedly large range of applications based on their various properties, such as antimicrobial, anticancer, or antioxidant [2]. These biological activities depend on the chemical composition, which is primarily determined by the plant genotype, but also greatly influenced by numerous factors such as geographical origin or environmental and agronomic conditions [3,4]. In addition, these chemical changes are strongly induced by the growth stage, which leads to the optimization of the harvest time [2,5]. Thus, research nowadays has been focused on the factors contributing to this variety, which has led to different chemotypes and chemical races being described [1,5,6].

*Foeniculum vulgare* Miller (FV, fennel), belonging to the Apiaceae family, is a glabrous, erect, glaucous green biennial or perennial plant that grows up to 2.5 m. Shiny and striate stems bear leaves that are more or less triangular in outline with filiform and acuminate lobes, while terminal umbels, formed by 12–25 tiny yellow flowers, give ovoid-oblong, sweet-tasting schizocarpic fruits up to 10 mm long [7]. It develops better in a mild climate, especially near the sea coast or on riverbanks, and grows better in drained, light, dry soil, with low acidity [8]. A wide range of bioactive compounds from this plant have been studied, with phenols, phenolic glycosides, and volatile aroma compounds, such as anethole, estragole, and fenchone, being reported as its major phytoconstituents [9]. The essential oil from fennel (FVEO) is often used as a flavoring agent but also as a constituent of various cosmetic and pharmaceutical products [10]. Numerous studies have been conducted to investigate FVEO’s composition from plant materials of different origins and have shown that the major constituents are phenylpropanoid derivatives and monoterpenoids [11,12]. In the majority of literature sources, the distillation process is restricted to 3 or 4 h. However, a recent report pointed out the importance of extending the duration up to 6 h, thus greatly influencing the FVEO composition and extraction yield, as well as biological activity [2,4]. Following that study, a fractionated extraction procedure was applied to the fennel fruit material collected in Montenegro. Liquid and vapor phases of essential oil (EO) samples were analyzed by Gas Chromatography/Mass Spectrometry (GC/MS) and Headspace-Gas Chromatography/Mass Spectrometry (HS-GC/MS) techniques.

## 2. Results

### 2.1. EO Extraction

Similarly as reported [4], plant material was collected in the fruiting stage normally associated with the richest EO content, and the extraction process lasted 6 h, as it was noted that the significant amount of EO was obtained after a three-hour distillation process. Thus, a total of 12 samples were obtained with three one-hour fractions (0 to 1, 1 to 2, and 2 to 3 h) and one three-hour fraction (3 to 6 h) for each of the three localities (Table 1).

**Figure 1:**
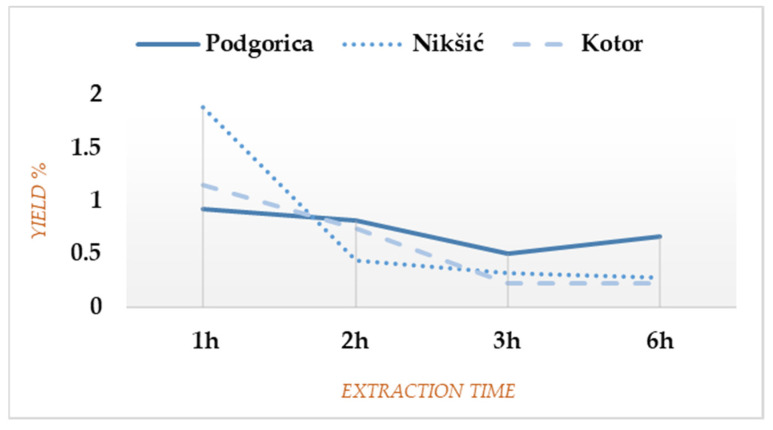
Yield curves for *Foeniculum vulgare* Miller (FV) from three Montenegrin localities.

Relative yield percentages calculated per weight of dried plant material for each EO fraction and cumulative yields over the entire extraction time are shown in Table S1 (Supplementary Materials). Total yields for the localities in Nikšić and Podgorica were quite similar (2.92% and 2.9%, respectively), whereas the material from Kotor gave 2.33% of EO. The biggest EO amount was observed during the first hour of each extraction (Figure 1), particularly for Nikšić (1.88% or even 64.38% of the total yield). After 1 h of the extraction process, a gradual decrease in yield was noted, except for Podgorica where the last fraction was still abundant (0.66% or 22.76% of the total yield), being higher than the third one, and up to 3 times higher than the corresponding ones for Nikšić and Kotor (9.59% and 9.44% of the total yields, respectively).

### 2.2. EO Chemical Composition

The chemical analyses of 12 EO samples obtained from plant material collected from three Montenegrin localities revealed the presence of 18 chemical constituents identified by GC/MS and HS-GC/MS techniques (Table 2, Table 3 and Table 4). Although both quantitative and qualitative differences in the samples were quite noticeable, phenylpropanoids anethole (18, ANE) and estragole (16, EST) with monoterpenoids *α*-terpineol (17, TER) and fenchone (12, FEN) could be singled out as the main compounds (Table S2, Supplementary Materials).

A total of 13 compounds was identified for the EO samples from Podgorica (Table 2, F1–F4 and F1HS–F4HS), with ANE, EST, and FEN as the most abundant ones. However, the ANE predominance was evident, particularly when the analysis was performed on the liquid phase. Its gradually increase during the time of extraction from 82.7% in the first hour (F1) to 89.5% in the last fraction (F4) was also noticeable. In contrast to that, FEN and EST contents gradually diminished during the duration (from 9.1% to 3.4% and from 6.4% to 5.1%, respectively). The vapor phase was characterized by a decreased ANE content and a slightly increased EST content (from 38.2% to 53.5% and from 6.4% to 10.4%, respectively). On the contrary, FEN was much more abundant than in the liquid phase with the biggest amount between the first and the second hours (32.7%, F2HS) and the smallest in the last fraction (15.9%, F4HS). However, no regularity in their content by fractions was observed.

The GC/MS analysis of the EO samples from Nikšić revealed the presence of 12 various constituents (Table 3, F5–F8) whereas the HS-GC/MS technique allowed the identification of 15 compounds (Table 3, F5HS–F8HS). Unlike the samples from Podgorica, the analysis of these ones showed the predominance of monoterpenoids FEN and TER, although still with a significant amount of ANE (from 22.8% to 30%). Whereas FEN content gradually diminished during the extraction time, from 30.9% in the first hour (F5) to 10.9% in the last 3 h (F8), TER was quite constantly abundant in each fraction (from 43.1% to 56.5%). The vapor phase analysis showed an increase in monoterpenoids content. Although more abundant in these samples, the FEN percentage gradually decreased with the extraction progress, from 62.0% (F5HS) to 29.1% (F8HS). On the contrary, TER increased from 20.9% in the first hour to 45.3% in the last fraction. A significantly smaller amount of ANE can be also observed in each fraction (up to 9.5%).

The EOs from Kotor were found to be composed of 11 and 13 compounds as identified by GC/MS and HS-GC/MS techniques, respectively (Table 4, F9–F12 and F9HS–F12HS). The analyses showed high similarity with the samples from Nikšić. Therefore, TER (from 32.1% to 42.2%) and FEN (from 7.1% to 17.8%) were the main monoterpenoid constituents, though phenylpropanoid ANE was more abundant than in the case of those samples (from 43.8% to 49.5%). The vapor phase was also characterized by the dominance of FEN and TER with a considerably smaller amount of ANE (up to 15.5%). Similar to the samples from Nikšić, the content of FEN decreased over the extraction time from 53.0% (F9HS) to 30.1% (F12HS), whereas the content of TER regularly increased from 23.1% in the first hour to 52.5% in the last 3 h of extraction.

Some of the constituents are characteristic only for the liquid phase, such as fenchyl acetate (13) and terpinen-4-ol (15) that were present in certain fractions of the EOs from each locality. However, their amounts were of low importance. On the other side, the headspace injection led to the identification of volatile compounds such as *α*-pinene (1), camphene (2), *β*-pinene (3), and *β*-phellandrene (7).

## 3. Discussion

The overall dynamic by which FV gives EO is quite different for every plant sample, although certain similarities could be observed for Nikšić and Kotor since the main part of those EOs was extracted within the first 2 h (79.44% and 81.96% of the total yields, respectively). On the contrary, the material from Podgorica formed an almost uniform yield curve until the end of the extraction process (Figure 1), which was partly overlapping with the data previously reported [4]. The results confirm there is no a priori rule on the extraction time for obtaining EOs [2]. The application of a standard three-hour distillation would have never highlighted the observed trend. For comparison purposes, a search on the new EO portal Py-EO under development by one of the authors (http://eo.3d-qsar.com, accessed on 23 November 2021) revealed the presence of 36 FVEO compositions. Discarding 18 compositions published in the previous article [4], most of the FVEO extractions were obtained by unfractionated hydro- and steam distillations with extraction times of 2, 3, 4, and 6 h and yields, ranging from 1.7 to 4.3%. No case was reported for FVEO from Montenegro. The FV plant materials were distilled either fresh or dried, and FVEO was extracted from different plant parts (fruits, leaves and full areal parts). In all listed EOs, the main constituents were found to be ANE, FEN, and EST (Table S3, Supplementary Materials).

ANT and its isomer EST (also known as methyl chavicol) are very common ingredients of FVEO, usually present in fruits and flowers, as reported in numerous papers [9,13,14,15,16]. These phenylpropanoids contribute a large component of the odor and flavor of many plants including *Pimpinella anisum* L. [17,18], *Anethum graveolens* L. [19], *Ocimum basilicum* L. [20,21], *Artemisia dracunculus* L. [22,23], and *Illicium verum* Hook.f. [19]. ANE is responsible for the sweet, distinct, anise-like flavor characterizing FV fruits [24]. It is reported to exhibit antimicrobial, anthelmintic, insecticidal, gastroprotective, antithrombotic, spasmolytic, antioxidant, anti-inflammatory, and antinociceptive properties [25,26,27]. Regarding the FVEO samples analyzed in this study, ANE can be considered as the most characterizing compound since it was present in each fraction from all the localities. It was recognized at its highest level for EO obtained from Podgorica strongly defining this chemotype (up to 89.5%). Moreover, it significantly determined the last fraction’s composition of each extraction. EST, on the other side, was characteristic of the samples from Podgorica, although never present in a large amount.

FEN is an irregular bicyclic monoterpene ketone. The same as ANE, it is one of the common constituents of FVEO [10,14,15,16]. Moreover, it is also a characterizing compound in the samples included in this study, always being present in a considerable amount. FEN diminished with the extraction progress; therefore, it is most abundant in its first 2 h. Comparing the localities, the higher FEN content for the samples from Nikšić and Kotor could be noted. As a source of bitterness in fennel, this monoterpene seems to be responsible for the antifungal and acaricidal activity of its EO [28], and it is reported to have analgesic [29] and anti-inflammatory properties [30]. It is a constituent of many other plants, including *Thuja*, *Lavandula,* and *Artemisia* species [31,32].

TER is a volatile monoterpenoid alcohol that occurs in a large number of EOs. It is one of five isomers, being the most common one found in nature, along with terpinen-4-ol. It is a very important constituent of several species, such as *Thymus caespititius* Brot. [33], *Salvia libanotica* Boiss. & Gaills [34], and *Melaleuca alternifolia* (Maiden & Betche) Cheel [35]. TER attracts a great interest as it has a wide range of biological applications as an antioxidant [36], anticancer [34], anticonvulsant [37], antiulcer [38], antihypertensive [39], anti-inflammatory [40], and antinociceptive compound [38]. It is frequently used in perfumes and cosmetics because of its pleasant odor [36]. The compound occurs only in the samples from Nikšić and Kotor, always among the main ones (up to 56.5%). To the best of the authors’ knowledge, this is the first report of such a high amount of TER. Along with FEN, it notably augments the fraction of monoterpenoids in these samples. In this regard, the opposite evolution (with the extraction development) of these two compounds can be observed, although always with the prevalence of TER (particularly evident in the second half of extraction).

The literature survey has revealed plenty of data. As a well-known industrial aromatic plant with different food and pharmaceutical applications, FV is thoroughly investigated. Thus, much research has been conducted so far to investigate its chemical composition. The results differ greatly depending on harvest time, region, and plant part, among other factors [4]. Some authors pointed out the FVEO composition is mostly dependent on the maturation period [41,42]. Generally, phenylpropanoid derivatives ANE and EST and monoterpenoids *α*-phellandrene, *α*-pinene, limonene, and FEN are usually reported as the main characterizing compounds [4,10,12,13,15,16,43,44]. The prevalence of monoterpenoids over the fraction of phenylpropanoids in the vegetative parts of FV can be considered as some general characteristic [15,16,45]. Contrary to that, EO obtained from fennel fruits is commonly characterized by the prevalence of the phenylpropanoid fraction, the presence of which is a stable characteristic, not dependent on origin [14,46]. The authors distinguished three intraspecific chemovarieties: FEN-rich, ANE-rich and EST-rich. Another study resulted in two different fruit-oil chemotypes: FEN-EST and FEN-ANE [43]. Phenylpropanoid content appeared to be associated with varieties, since *azoricum* and *dulce* were found to contain mostly ANE, while EST was found in prevalence in the *vulgare* variety [47]. All these analyses are in accordance with the results obtained for the samples from Podgorica (F1–F4) presented herein (Table 2). Undoubtedly, the FVEO from Podgorica belongs to the ANE chemotype. In addition, a comparison with the results from the previous report showed certain similarities [4]. In that study, a 24-h fractionated steam distillation procedure was applied to the FV material from Italy (Tarquinia, Viterbo) and was monitored for 3 months, including vegetative and reproductive stages. A great increase in EO yield (up to 5 times) was noticed in October (fruiting material, the only one comparable with the results presented herein), as well as a substantial difference in EO composition. EST was identified as the highest-level main constituent for those EO samples, being particularly abundant in the first 6 h (up to 57.6%), accompanied by a significant amount of FEN (up to 14.1%). A gradual decrease with the extraction duration was observed for both of these compounds. This trend can be equated with FVEOs from Podgorica, although the EST content in the samples from Italy was drastically larger.

Chemical profiles of the EO samples from Nikšić (F5-F8) and Kotor (F9–F12), however, cannot be related to any of those already reported (Table 3 and Table 4); this is primarily because of the high amount of TER (from 32.1% to 56.5%). This monoterpenoid, always accompanied by the lower amount of FEN, is combined with a significant amount of the phenylpropanoid ANE (up to 49.5%). Still, there is a slight difference between these localities: Whereas the samples from Kotor are quite rich in ANE, the ones from Nikšić are characterized by the prevalence of the monoterpene fraction. Accordingly, a new chemotype from Montenegro rich in TER can be defined.

Intraspecific chemical polymorphism is quite common in aromatic plants. It depends on a combination of factors related to the environment and genetics, as well as the anatomical and physiological characteristics of plants. These factors are difficult to verify, so the existence of different chemotypes is often not clearly related to the possible causes [5]. Keeping in mind the significant climatic and geographic differences between the three selected localities, a certain relationship between chemical variations and habitats can be suggested. However, additional ecological and eco-physiological analyses are needed.

The study presented herein also included the vapor phase analysis. The samples were characterized by an increase in the monoterpene fraction, mainly represented by FEN and TER. The percentages of FEN were particularly higher: Up to 4 times than the ones reported in the liquid phases, even 5 times in some fractions of FVEO from Podgorica (F3HS). However, some other minor compounds enhanced their amounts with vaporizing, such as *α*-phellandrene (5, up to 5.5%), limonene (6, up to 6.4%), 1,8-cineole (8, up to 5.2%), and *γ*-terpinene (9, up to 7.1%). Whereas the samples from Podgorica were still abundant in phenylpropanoids (with ANE being in prevalence over EST), the ones from Nikšić and Kotor were significantly deprived of this fraction.

A search of the literature available has revealed little data regarding this type of analysis. However, while FVEO’s vapor phase has not been analyzed, the headspace aroma of certain FV organs has. Thus, an analysis on the *azoricum* variety included bulbs and aerial parts material from Egypt, Spain, and Holland. The analysis showed that monoterpenes prevailed in most headspace matrices, with an abundance of 6 (up to 61.54%) followed by a significant amount of ANE, particularly in the Egyptian samples [48]. Further, another study included a comprehensive aroma profiling amongst FV fruit accessions of various origins reporting the predominance of ANE, particularly in the *vulgare* variety. Additionally, an *azoricum* variety accession from Austria was rich in EST and FEN as well [24].

The volatile analysis of the FVEO samples presented herein revealed chemical profiles quite different from the corresponding ones in the liquid phases, generally with much higher amounts of low-boiling components and smaller amounts of the heaviest ones (Figures S1–S6, Supplementary Materials). Moreover, the headspace injection allowed the identification of some constituents (**1**, **2**, **3,** and **7**) not found with the liquid phase analysis, thus highlighting the capability of this technique of minor compounds detection. To the best of the authors’ knowledge, this analysis has been the pioneer for FVEO.

Bearing in mind the great influence of the chemical composition on the biological properties, as well as the effects of synergism and/or antagonism between the main and/or minor compounds, various further investigations can be suggested. In that sense, FVEO samples from Nikšić and Kotor abundant in TER have a priority of importance due to the numerous biological applications of this monoterpenoid.

## 4. Materials and Methods

### 4.1. Plant Material

Ripe fruits of wild growing FV were collected from 3 localities in Montenegro covering the Mediterranean and sub-mediterranean regions: Doclea (42°28′05.1″ N, 19°16′03.1″ E), a suburban archeological site about 3 km from Podgorica city, Uzdomir hill (42°48′09.7″ N, 18°55′24.8″ E) in the rural area of Nikšić, and the foothill of the St. John’s Fortress in Kotor (42°25′35.2″ N, 18°46′36.8″ E), approximately 700 m from the seacoast. The FV material was collected in the early autumn period of 2019. Air-drying of the collected material was performed in a shady place for approximately 20 days. Voucher specimens have been deposited in the Department of Biology at the University of Montenegro (numbers BDPMF-FV/01, BDPMF-FV/02, and BDPMF-FV/03). Taxonomic identification of the species was conducted according to the official European flora [7].

### 4.2. EOs Extraction

EOs have been isolated by hydrodistillation using a Clevenger-type apparatus with the extraction method previously reported [2,4]. Dried fennel fruits in toto (50 g) were subjected to fractionated distillation, collecting EOs at interval times of 1, 2, 3, and 6 h. At each interval, the accumulated oil/water double phase was extracted 3 times with 20 mL of diethyl ether, then the organic layers were dried over anhydrous sodium sulfate (Na_2_SO_4_), filtered, and deprived of the solvent in vacuo to furnish EOs. Thus, 4 fractions from each FV sample were obtained, and the prepared EOs were stored in tightly closed dark vials until further analysis. 

### 4.3. EO Analysis

A chemical analysis of FVEO samples was performed for both liquid and vapor phases. The analyses were carried out twice, showing reproducible results.

#### 4.3.1. GC/MS Analysis

For the chemical quali-quantitative analysis of the EO samples, a gas chromatograph (GC) directly coupled to a mass spectrometer (MS) Perkin Elmer Clarus 500 model was used. The GC was equipped with two columns, one of which was a Restek Stabilwax (fused-silica) polar capillary column, and the other was a Varian (VF-1ms) apolar column. Helium was used as the carrier gas (1.0 mL/min). The column temperature was programmed as follows: from 60 °C to 220 °C at a rate of 5 °C/min, and held for 10 min. The MS parameters were ionization voltage taken at 70 eV, the mass range was from 40 to 500 *m/z*, the ion source temperature of 200 °C, and a scan time of 0.2 s.

The identification of the components separated by GC/MS was performed first by comparing the mass spectra for each compound with that reported in the MS libraries database (Wiley and Nist 02) and then by comparison of Linear Retention Indices (LRI) of each compound calculated using a mixture of n-alkanes (C8–C30 aliphatic hydrocarbons, Ultrasci, injected into both polar and apolar columns under the same operating conditions), with available retention indices in the literature. GC-FID (flame-ionization detector) analysis was performed under the same experimental conditions using the polar column as described for the GC-MS measurements. Relative percentages for quantification of the components were calculated by electronic integration of the GC-FID peak areas using the normalization method without using corrections factors (RRFs).

#### 4.3.2. HS-GC/MS Analysis

To describe the vapor phase chemical profile, a Perkin-Elmer Headspace Turbomatrix 40 autosampler connected to a Clarus 500 GC-MS was used. About 2 mL of the sample was placed in a 20 mL vial sealed with headspace PTFE-coated silicone rubber septa and caps. The headspace parameters applied were the following: Needle temperature was 90 °C; 3.5 min pressurization time; and 0.3 min of injection time. The gas phase of the sealed vials was equilibrated for 10 min at 60 °C and was followed immediately by compound desorption into the GC injector in splitless mode. Quantification of the compounds was performed by GC-FID under the same conditions described above.

## 5. Conclusions

In line with the previous studies [2,4], a fractionated hydrodistillation extraction procedure was applied to the ripe FV fruit material collected from three Montenegrin localities: Podgorica, Nikšić, and Kotor. The obtained FVEO samples were analyzed by GC/MS and HS-GC/MS to characterize the volatile chemical composition of both liquid and vapor phases. The analyses conducted revealed the existence of a new, TER-rich chemotype previously unreported.

The extraction method applied gave FVEO fractions that differ greatly in their chemical compositions. Although the main characterizing constituents are always present, variations in their amount are particularly evident between the first three fractions (up to 3 h of extraction) and the last one (the last 3 h of extraction). Whereas the material from Podgorica gave EOs belonging to the well-known ANE-rich chemotype, the ones from Nikšić and Kotor were characterized by the predominance of a monoterpenoid fraction. This chemotype is particularly rich in TER, also containing significant amounts of FEN and ANE, the common FVEO ingredients. Regarding the individual fractions of the FVEO samples from each locality, the results obtained are in compliance with the previous work [4], indicating the importance of hydrodistillation extension after the last fraction, between the third and the sixth hours, which can greatly contribute to the total yield and composition.

EO vapor phases were more enriched in monoterpenoids, with FEN being the most abundant one accompanied by a significant amount of TER. A markedly decreased amount of phenylpropanoids was observed in all of the samples as well. Therefore, low-boiling terpenes are more abundant when using headspace injection. The use of both analytical techniques represented valid applicability for better identification of the FVEO components.

## Figures and Tables

**Table 1:** A total of 12 essential oil (EO) samples obtained for each of the 3 localities.

Sample Name ^1^		Time of Extraction
liquid phase	vapor phase	
Podgorica		
F1	F1HS	from 0th to 1st h
F2	F2HS	from 1st to 2nd h
F3	F3HS	from 2nd to 3rd h
F4	F4HS	from 3rd to 6th h
Nikšić		
F5	F5HS	from 0th to 1st h
F6	F6HS	from 1st to 2nd h
F7	F7HS	from 2nd to 3rd h
F8	F8HS	from 3rd to 6th h
Kotor		
F9	F9HS	from 0th to 1st h
F10	F10HS	from 1st to 2nd h
F11	F11HS	from 2nd to 3rd h
F12	F12HS	from 3rd to 6th h

^1^ F–Fennel, 1–12–Ordinal number of the sample, HS–Head space analysis.

**Table 2:** Chemical composition (%) of FV essential oils (FVEOs) from Podgorica analyzed by GC/MS and HS-GC/MS.

N°	Component ^1^	LRI^lit 2^	LRI ^3^	LRI ^4^	F1	F1HS	F2	F2HS	F3	F3HS	F4	F4HS
1	*α*-pinene	1021	1018	933	-	-	-	1.5	-	0.5	-	2.4
2	camphene	1063	1060	944	-	-	-	-	-	-	-	0.2
3	*β*-pinene	1108	1100	980	-	-	-	-	-	-	-	0.2
4	*β* -myrcene	1160	1155	984	-	0.5	0.1	1.7	-	1.7	0.2	3.4
5	*α*-phellandrene	1177	1173	1004	0.2	3.4	0.3	4.5	0.2	3.5	0.3	5.5
6	limonene	1198	1200	986	0.1	2.0	0.3	3.7	0.3	3.9	0.4	6.4
7	*β*-phellandrene	1205	1206	1026	-	-	-	-	-	-	-	0.5
8	1,8-cineole	1209	1211	998	0.2	1.6	0.4	4.5	0.3	4.0	0.4	5.2
9	*γ*-terpinene	1243	1242	1071	0.1	1.7	0.4	3.6	0.4	4.7	0.6	7.1
10	p-cymene	1268	1271	1030	0.2	2.4	0.1	1.3	-	0.8	-	1.1
11	terpinolene	1282	1284	1078	-	-	-	0.2	-	0.2	-	0.4
12	fenchone	1422	1420	1082	9.1	24.5	7.5	32.7	5.1	26.8	3.4	15.9
13	fenchyl acetate	1470	1465	1210	0.2	-	0.1	-	-	-	-	-
14	camphor	1507	1501	1135	0.2	-	0.2	0.5	0.1	0.4	0.1	0.2
15	terpinen-4-ol	1603	1601	1175	0.6	-	-	-	-	-	-	-
16	estragole	1655	1658	1178	6.4	10.4	6.1	7.6	5.8	7.7	5.1	6.4
17	*α*-terpineol	1729	1730	1190	-	-	-	-	-	-	-	-
18	anethole	1837	1840	1260	82.7	53.5	84.5	38.2	87.8	45.7	89.5	45.1
Total (%)					100.0	100.0	100.0	100.0	100.0	99.9	100.0	100.0
Monoterpenoids					9.3	26.1	7.9	37.2	5.4	30.8	3.8	21.1
Monoterpenes					0.3	4.6	0.6	6.8	0.4	7.4	0.8	12.0
Monoterpenes alcohol					0.6	-	-	-	-	-	-	-
Monoterpenes cyclic					0.1	5.4	0.6	9.7	0.5	7.9	0.7	15.2
Others					89.5	63.9	90.9	46.3	93.7	53.8	94.7	51.7

^1^ Elution order on polar column. ^2^ Linear Retention indices from literature. ^3^ Linear Retention indices measured on polar column. ^4^ Linear Retention indices measured on apolar column. -: Traces < 0.1%.

**Table 3:** Chemical composition (%) of FVEOs from Nikšić analyzed by GC/MS and HS-GC/MS.

N°	Component ^1^	LRI^lit 2^	LRI ^3^	LRI ^4^	F5	F5HS	F6	F6HS	F7	F7HS	F8	F8HS
1	*α*-pinene	1021	1018	933	-	2.3	-	0.2	-	0.3	-	0.1
2	camphene	1063	1060	944	-	0.5	-	0.1	-	0.1	-	-
3	*β*-pinene	1108	1100	980	-	0.1	-	-	-	-	-	-
4	*β*-myrcene	1160	1155	984	0.4	1.9	0.2	1.7	0.3	2.7	0.3	2.9
5	*α*-phellandrene	1177	1173	1004	0.2	1.0	0.9	0.7	0.1	0.8	-	0.8
6	limonene	1198	1200	986	0.5	2.4	0.6	3.4	0.8	5.2	0.9	6.1
7	*β*-phellandrene	1205	1206	1026	-	0.4	-	-	-	0.2	-	0.1
8	1,8-cineole	1209	1211	998	0.6	2.7	0.4	2.2	0.4	2.5	0.4	2.3
9	*γ*-terpinene	1243	1242	1071	0.2	0.9	0.4	1.5	0.4	2.2	0.5	2.5
10	p-cymene	1268	1271	1030	0.2	0.9	-	0.3	0.1	0.3	-	0.3
11	terpinolene	1282	1284	1078	-	0.1	-	0.2	0.1	0.3	0.1	0.5
12	fenchone	1422	1420	1082	30.9	62.0	19.2	50.6	16.9	43.0	10.9	29.1
13	fenchyl acetate	1470	1465	1210	0.2	-	-	-	-	-	-	-
14	camphor	1507	1501	1135	0.8	0.6	0.6	0.5	0.4	0.4	0.2	0.3
15	terpinen-4-ol	1603	1601	1175	-	-	0.1	-	0.1	-	0.2	-
16	estragole	1655	1658	1178	-	-	-	-	-	-	-	-
17	*α*-terpineol	1729	1730	1190	43.1	20.9	49.6	32.9	53.9	36.5	56.5	45.3
18	anethole	1837	1840	1260	22.8	3.3	28.0	5.4	26.5	5.5	30.0	9.5
Total (%)					99.9	100.0	100.0	99.7	100.0	100.0	100.0	99.8
Monoterpenoids					31.5	64.7	19.6	52.8	17.3	45.5	11.3	31.4
Monoterpenes					0.7	3.8	0.6	3.7	0.9	5.5	0.9	6.2
Monoterpenes alcohol					43.1	20.9	49.7	32.9	54.0	36.5	56.7	45.3
Monoterpenes cyclic					0.3	6.7	1.5	4.4	0.9	6.6	0.9	7.1
Others					23.8	3.9	28.6	5.9	26.9	5.9	30.2	9.8

^1^ Elution order on polar column. ^2^ Linear Retention indices from literature. ^3^ Linear Retention indices measured on polar column. ^4^ Linear Retention indices measured on apolar column. -: Traces < 0.1%.

**Table 4:** Chemical composition (%) of FVEOs from Kotor analyzed by GC/MS and HS-GC/MS.

N°	Component ^1^	LRI^lit 2^	LRI ^3^	LRI ^4^	F9	F9HS	F10	F10HS	F11	F11HS	F12	F12HS
1	*α*-pinene	1021	1018	933	-	0.9	-	2.2	-	-	-	-
2	camphene	1063	1060	944	-	-	-	-	-	-	-	-
3	*β*-pinene	1108	1100	980	-	-	-	-	-	-	-	-
4	*β*-myrcene	1160	1155	984	0.1	1.0	0.4	2.1	-	1.1	-	1.3
5	*α*-phellandrene	1177	1173	1004	0.2	1.9	0.4	3.2	0.1	1.1	-	1.0
6	limonene	1198	1200	986	0.3	2.1	0.8	4.5	0.3	2.5	0.3	3.6
7	*β*-phellandrene	1205	1206	1026	-	0.4	-	0.6	-	0.1	-	0.1
8	1,8-cineole	1209	1211	998	0.5	3.3	0.7	3.9	0.2	1.9	0.2	2.1
9	*γ*-terpinene	1243	1242	1071	0.2	1.1	0.5	2.5	0.3	2.1	0.3	2.8
10	p-cymene	1268	1271	1030	0.3	1.7	0.1	0.6	-	0.4	-	0.4
11	terpinolene	1282	1284	1078	-	0.1	-	0.2	-	0.3	-	0.4
12	fenchone	1422	1420	1082	17.8	53.0	16.4	42.4	10.7	34.4	7.1	30.1
13	fenchyl acetate	1470	1465	1210	0.2	-	0.1	-	0.1	-	-	-
14	camphor	1507	1501	1135	0.5	0.6	0.4	0.4	0.3	0.4	0.2	0.3
15	terpinen-4-ol	1603	1601	1175	-	-	-	-	0.1	-	0.1	-
16	estragole	1655	1658	1178	-	-	-	-	-	-	-	-
17	*α*-terpineol	1729	1730	1190	32.1	23.1	36.1	26.9	41.5	40.2	42.2	52.5
18	anethole	1837	1840	1260	47.7	10.7	43.8	10.5	46.4	15.5	49.5	5.4
Total (%)					99.9	99.9	99.7	100.0	100.0	99.9	99.9	100.0
Monoterpenoids					18.3	56.3	17.5	46.3	10.9	36.3	7.3	32.2
Monoterpenes					0.6	3.9	1.0	5.4	0.3	3.9	0.3	4.9
Monoterpenes alcohol					32.1	23.1	36.1	26.9	41.6	40.2	42.3	52.5
Monoterpenes cyclic					0.5	5.3	1.2	10.5	0.4	3.7	0.3	4.7
Others					48.2	11.3	44.4	10.9	46.8	15.9	49.7	5.7

^1^ Elution order on polar column. ^2^ Linear Retention indices from literature. ^3^ Linear Retention indices measured on polar column. ^4^ Linear Retention indices measured on apolar column. -: Traces < 0.1%.

## Data Availability

Not applicable.

## Supplementary Materials

The following are available online at https://www.mdpi.com/article/10.3390/plants11010042/s1, Figure S1: Trend of major compounds for F1–F4 samples, Figure S2: Trend of major compounds for F5-F8 samples, Figure S3: Trend of major compounds for F9–F12 samples, Figure S4: Trend of the low-boiling compounds for F1–F4 samples, Figure S5: Trend of the low-boiling compounds for F5-F8 samples, Figure S6: Trend of the low-boiling compounds for F9–F12 samples, Figure S7: Doclea, Podgorica; natural habitat of *Foeniculum vulgare* Miller (FV), Figure S8: Uzdomir, Nikšić; natural habitat of FV, Figure S9: St. John’s Fortress, Kotor; natural habitat of FV, Table S1: Yield% of FV from Montenegro, Table S2: Most characterizing components of FV essential oils (FVEOs) from Montenegro: chemical structures, MWs and CAS numbers, Table S3: Chemical composition (%) of various FVEOs as listed in the http://eo.3d-qsar.com site (accessed on 23 November 2021).
