# Supplementary material for: Foeniculum vulgare Miller, a New Chemotype from Montenegro
Source: Plants (Basel). 2021 Dec 23;11(1):42. doi: 10.3390/plants11010042 (PMC8747267; doi:10.3390/plants11010042)
Supplement: Supplementary file 1 [file plants-11-00042-s001.zip › plants-1500533-supplementary.pdf]

Supplementary Materials

Foeniculum vulgare Miller, a new chemotype from Montenegro

Mijat Božović[1,¥], Stefania Garzoli[2,¥], Svetlana Vujović[3], Filippo Sapienza[2], Rino Ragno[2]

Affiliation

[1] Faculty of Natural Sciences and Mathematics, University of Montenegro, Podgorica, Montenegro
[2] Department of Drug Chemistry and Technology, Sapienza University, Rome, Italy
[3] Institute for Medicines and Medical Devices in Montenegro, Podgorica, Montenegro

Correspondence

Dr. Mijat Božović
Faculty of Natural Sciences and Mathematics
University of Montenegro
Podgorica, Montenegro
Tel.: +382-20-243-816
Fax: +382-20-244-608
mijatboz@ucg.ac.me

Dr. Rino Ragno
Department of Drug Chemistry and Technology
Sapienza University
Rome, Italy
Tel.: +39-6-4991-3937
Fax: +39-6-4991-3627

rino.ragno@uniroma1.it

¥ Contributed equally to the paper

Contents:

Figure S1: Trend of major compounds for F1-F4 samples.

Figure S2: Trend of major compounds for F5-F8 samples.

Figure S3: Trend of major compounds for F9-F12 samples.

Figure S4: Trend of the low-boiling compounds for F1-F4 samples.

Figure S5: Trend of the low-boiling compounds for F5-F8 samples.

Figure S6: Trend of the low-boiling compounds for F9-F12 samples.

Figure S7: Doclea, Podgorica; natural habitat of *Foeniculum vulgare* Miller (FV) (photo by Svetlana Vujović).

Figure S8: Uzdomir, Nikšić; natural habitat of FV (photo by Svetlana Vujović).

Figure S9: St. John's Fortress, Kotor; natural habitat of FV (photo by Svetlana Vujović).

Table S1: Yield % of FV from Montenegro.

Table S2: Most characterizing components of FV essential oils (FVEOs) from Montenegro: chemical structures, MWs and CAS numbers.

Table S3: Chemical composition (%) of various FVEOs as listed in the http://eo.3d-qsar.com site.

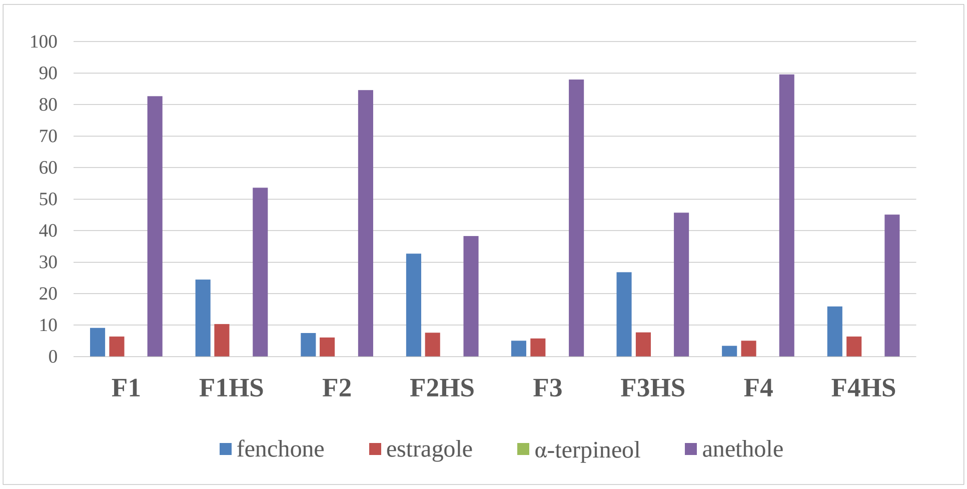

Figure S1: Trend of major compounds for F1-F4 samples.

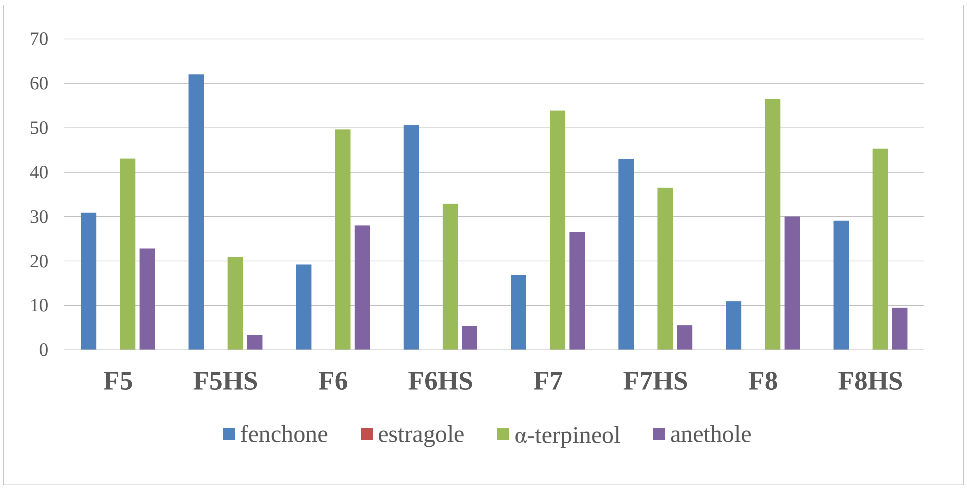

Figure S2: Trend of major compounds for F5-F8 samples.

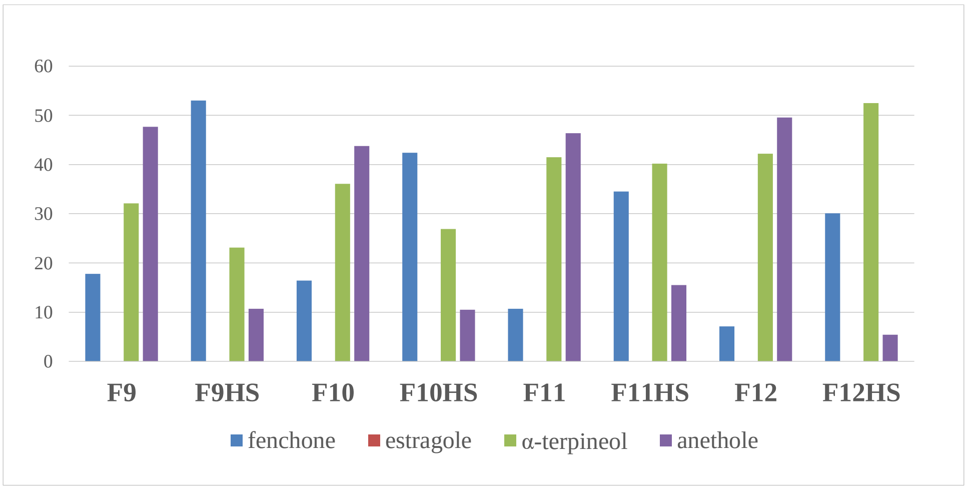

Figure S3: Trend of major compounds for F9-F12 samples.

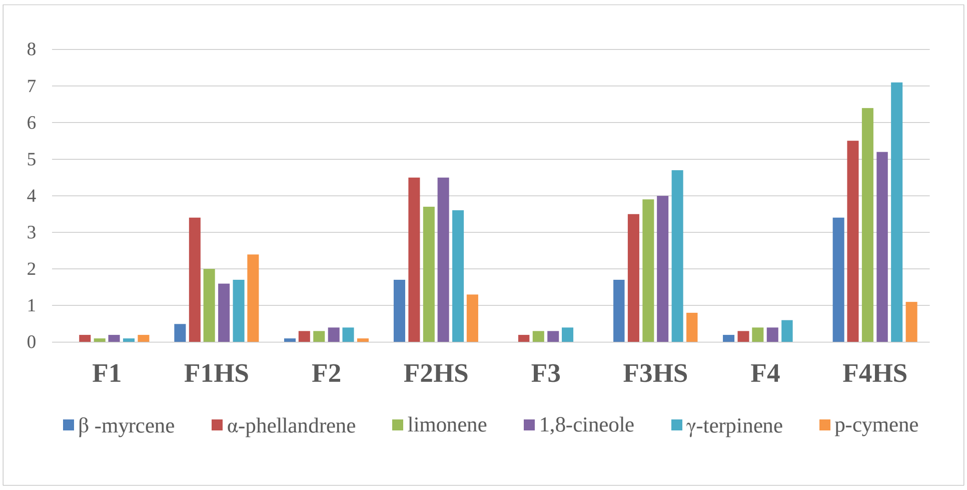

Figure S4: Trend of the low-boiling compounds for F1-F4 samples.

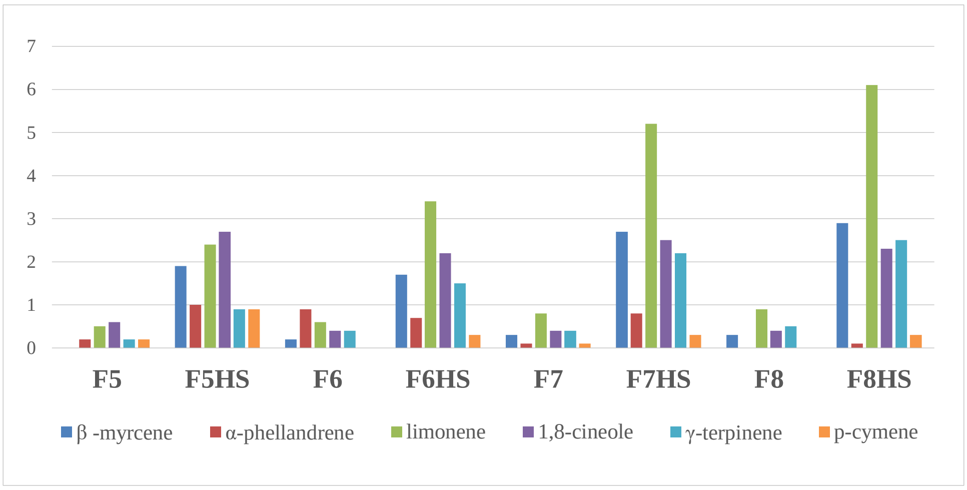

Figure S5: Trend of the low-boiling compounds for F5-F8 samples.

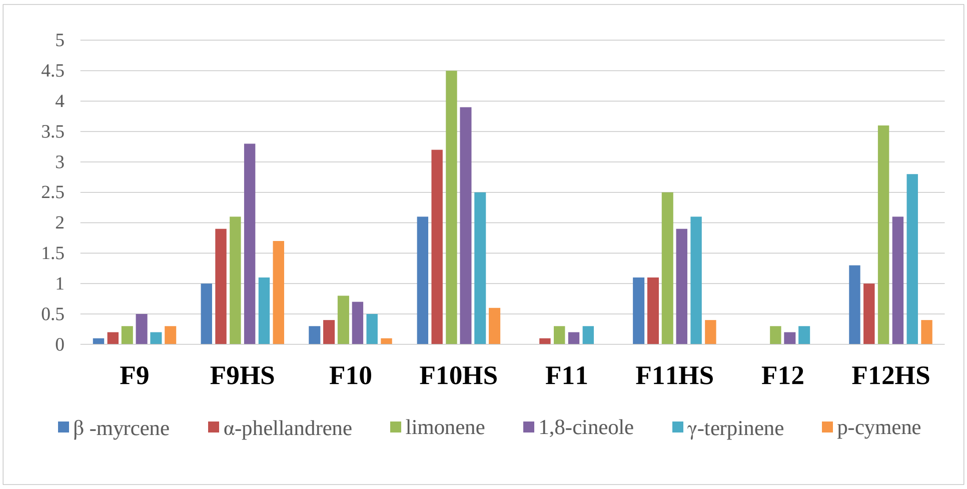

Figure S6: Trend of the low-boiling compounds for F9-F12 samples.

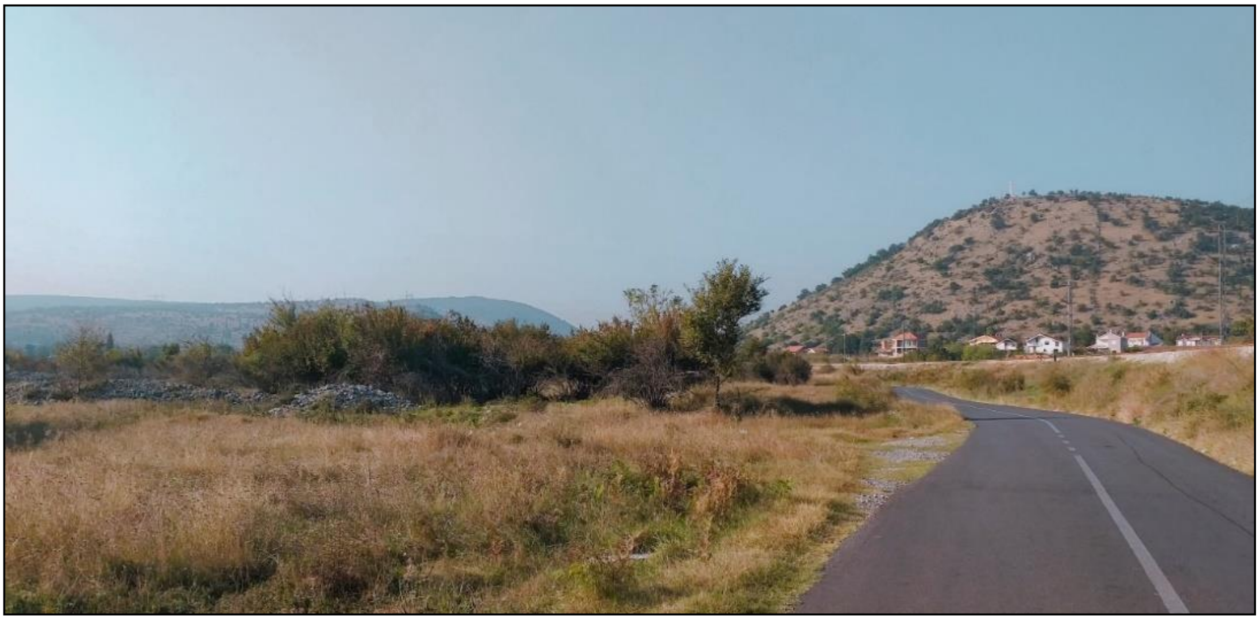

Figure S7: Doclea, Podgorica; natural habitat of *Foeniculum vulgare* Miller (FV) (photo by Svetlana Vujović).

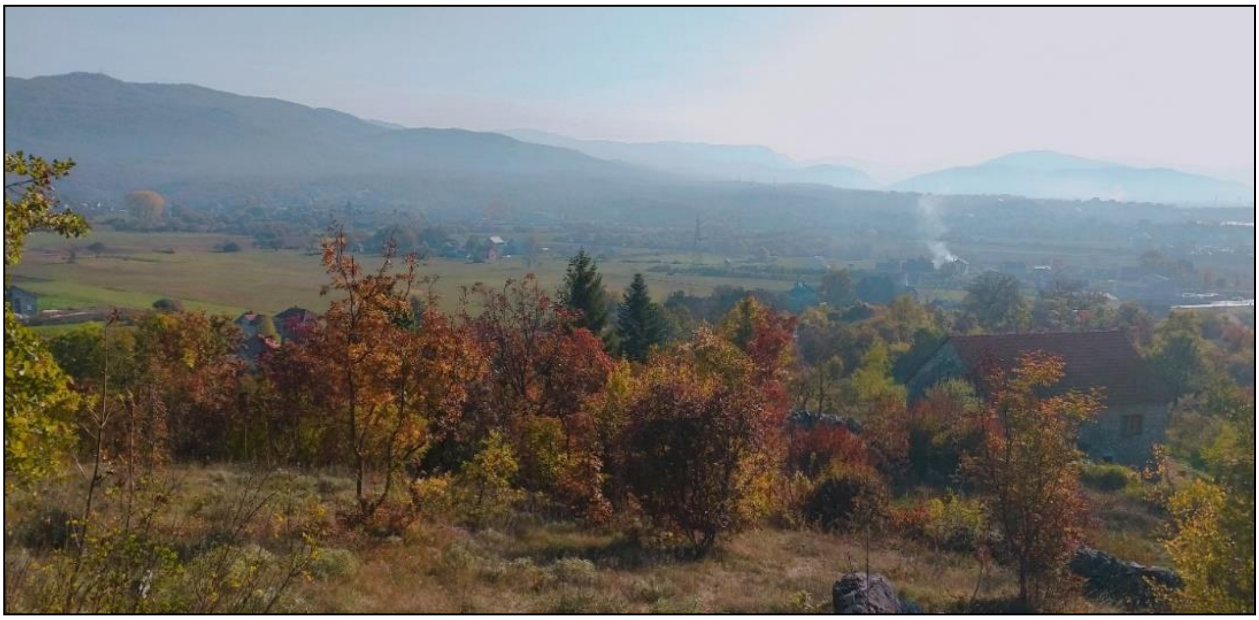

Figure S8: Uzdomir, Nikšić; natural habitat of FV (photo by Svetlana Vujović).

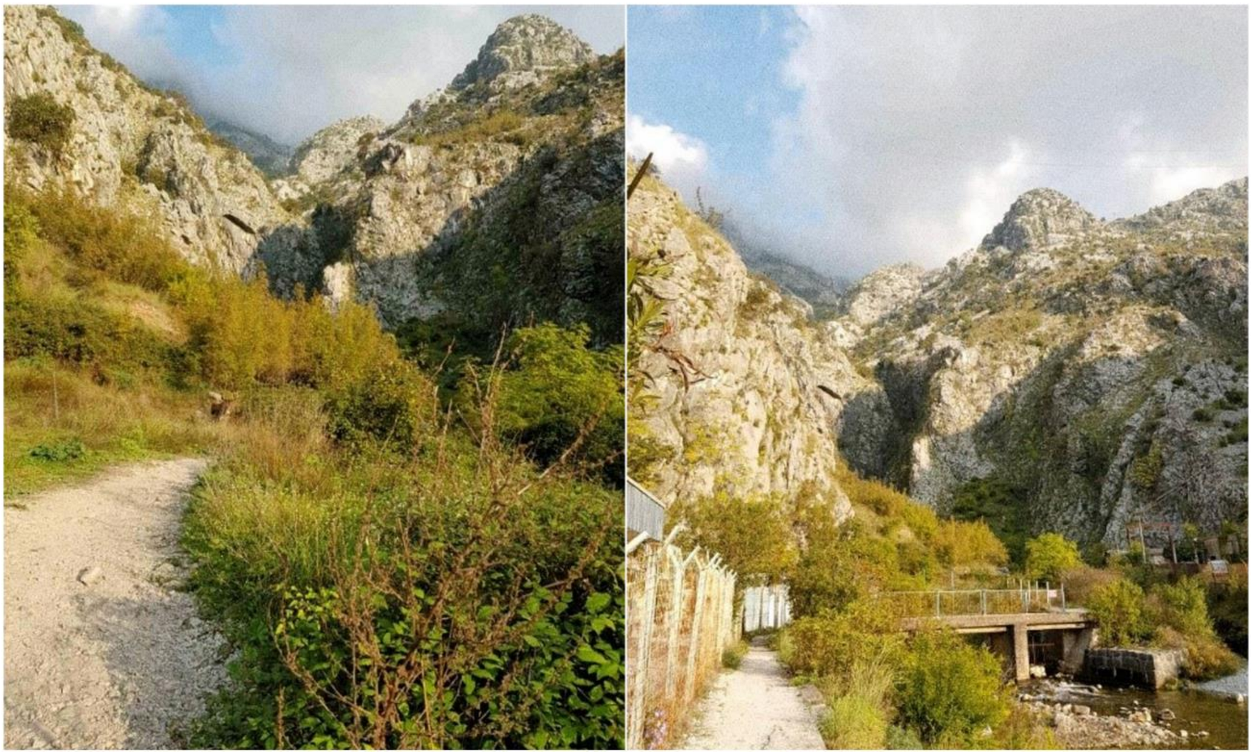

Figure S9: St. John's Fortress, Kotor; natural habitat of FV (photo by Svetlana Vujović).

Table S1: Yield % of FV from Montenegro.

	extraction time											
	**1h**			**2h**			**3h**			**6h**		
locality	**y** [a]	**cy** [b]	**s** [c]	**y** [a]	**cy** [b]	**s** [c]	**y** [a]	**cy** [b]	**s** [c]	**y** [a]	**cy** [b]	**s** [c]
**Podgorica**	0.92	**0.92**	***31.72***	0.82	**1.74**	***28.27***	0.5	**2.24**	***17.24***	0.66	**2.9**	***22.76***
**Nikšić**	1.88	**1.88**	***64.38***	0.44	**2.32**	***15.06***	0.32	**2.64**	***10.95***	0.28	**2.92**	***9.44***
**Kotor**	1.15	**1.15**	***49.35***	0.74	**1.86**	***32.61***	0.22	**2.11**	***9.44***	0.22	**2.33**	***9.59***

[a] Yield % calculated on the dried FV plant material. [b] Cumulative yield % of FVEOs over time. [c] Shared % of the total yield.

Table S2: Most characterizing components of FV essential oils (FVEOs) from Montenegro: chemical structures, MWs and CAS numbers.

# [1]	Chemical Structure	Name	MW	CAS
12	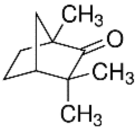	fenchone	152.23	7787-20-4
16	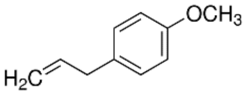	estragole	148.20	140-67-0
17	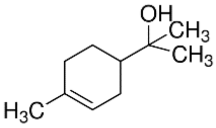	α-terpineol	154.25	98-55-5
18	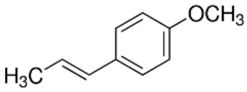	anethole	148.20	4180-23-8

[1] # indicates the compound identification number.

Table S3: Chemical composition (%) of various FVEOs as listed in the http://eo.3d-qsar.com site. Components displaying an average composition lower than 2 were omitted.

DOI	Extraction Type	Extraction Yield	Extraction Time	Plant Part	Plant State	Country	alpha-pinene	pha-phellandren	camphor	limonene	Fenchone	Estragole	anethole
10.1080/03235408.2013.853456	Hydro Distillation	3.5	NA	Seeds	Dry	Iran	0.0	2.7	0.0	0.0	9.4	3.5	75.2
10.2478/pjct-2019-0018	Hydro Distillation	4.18	2.0 h	Fruits	Fresh	Poland	2.9	0.0	0.3	2.3	18.1	2.6	70.0
10.1016/j.bjp.2015.02.009	Hydro Distillation	NA	3.0 h	Leaves	Fresh	Brazil	1.5	0.0	0.0	21.3	1.2	1.8	70.2
Progetto_OE_Commerciali	Commercial	NA	NA	NA	NA	NA	1.0	0.0	0.0	3.4	3.4	2.2	78.1
10.1016/j.biopha.2016.10.013	NA	NA	NA	NA	NA	NA	5.6	3.7	0.3	6.3	5.2	8.1	56.3
10.1080/14786419.2017.1292266	Hydro Distillation	2.2	3.0 h	Areal Part	Fresh	Portugal	10.1	11.0	0.1	3.6	10.8	4.0	47.0
10.1080/10412905.2015.1025919	Steam Distillation	NA	NA	Seeds	NA	NA	4.7	1.0	0.0	4.0	14.3	2.1	71.3
10.1007/s10340-016-0759-2	Hydro Distillation	NA	4.0 h	Fruits	Fresh	Poland	2.8	0.0	0.6	2.1	33.4	0.0	39.2
10.1371/journal.pone.0165667	Hydro Distillation	NA	4.0 h	Seeds	Dry	NA	1.1	0.0	0.0	10.5	4.6	80.8	1.0
10.3390/molecules23071549	Commercial	NA	NA	NA	NA	NA	3.6	0.5	0.1	11.5	6.5	0.6	75.1
10.1007/s11274-011-0939-4	Hydro Distillation	NA	NA	NA	Dry	NA	0.0	0.0	0.4	1.6	0.0	0.0	88.9
10.1016/j.indcrop.2012.10.012	Hydro Distillation	1.95	6.0 h	Seeds	Dry	Egypt	1.6	0.0	0.0	4.2	8.3	10.4	56.4
10.1016/j.indcrop.2019.111854	Hydro Distillation	3.6	3.0 h	Fruits	Dry	Serbia	0.5	0.2	0.5	1.1	22.6	2.7	69.9
10.1016/j.indcrop.2019.111854	Hydro Distillation	4	3.0 h	Fruits	Dry	Serbia	2.0	0.4	0.5	2.3	23.1	2.6	64.9
10.24193/subbchem.2019.2.11	Hydro Distillation	1.7	4.0 h	Seeds	Dry	Romania	0.0	0.0	0.0	7.3	1.3	1.3	89.6
10.1080/10412905.2016.1146169	Hydro Distillation	NA	2.0 h	Seeds	Dry	Iran	3.6	0.1	0.0	20.6	7.2	57.9	5.0
10.1016/j.indcrop.2016.02.064	Hydro Distillation	NA	3.0 h	Fruits	Dry	Romania	0.0	0.0	21.3	0.0	0.5	0.6	58.1
10.3109/09637486.2014.953454	Commercial	NA	NA	Areal Part	Flowering	Scotland	1.7	1.1	0.0	2.8	7.1	8.1	58.7
